# Long-Acting Cabotegravir/Rilpivirine Concentrations in Combination With Intravenous Rifampin: A Case Report

**DOI:** 10.1093/ofid/ofad604

**Published:** 2023-12-02

**Authors:** Shawnalyn W Sunagawa, Joshua P Havens, Anthony Podany, Bryan Walker, Kimberly K Scarsi, Sara H Bares

**Affiliations:** College of Pharmacy, University of Nebraska Medical Center, Omaha, Nebraska, USA; College of Pharmacy, University of Nebraska Medical Center, Omaha, Nebraska, USA; Division of Infectious Diseases, Department of Internal Medicine, College of Medicine, University of Nebraska Medical Center, Omaha, Nebraska, USA; College of Pharmacy, University of Nebraska Medical Center, Omaha, Nebraska, USA; Division of Infectious Diseases, Department of Internal Medicine, College of Medicine, University of Nebraska Medical Center, Omaha, Nebraska, USA; College of Pharmacy, University of Nebraska Medical Center, Omaha, Nebraska, USA; Division of Infectious Diseases, Department of Internal Medicine, College of Medicine, University of Nebraska Medical Center, Omaha, Nebraska, USA; Division of Infectious Diseases, Department of Internal Medicine, College of Medicine, University of Nebraska Medical Center, Omaha, Nebraska, USA

**Keywords:** drug-drug interactions, long-acting cabotegravir/rilpivirine, plasma concentrations, rifamycins

## Abstract

As antiretroviral therapy advancements focus on long-acting medications, there is a need to assess the potential impact of drug–drug interactions. We present a real-world case of long-acting cabotegravir/rilpivirine co-administered with intravenous rifampin. The combination resulted in both cabotegravir and rilpivirine concentrations falling below 4 times the protein-adjusted IC_90_.

Globally, the annual number of patients with tuberculosis (TB) has remained relatively unchanged; however, patients with HIV still account for a disproportionate number of TB-associated deaths [1]. Improved access to antiretroviral therapy (ART) has decreased the incidence of HIV-related TB. Previous studies have demonstrated improved outcomes with ART initiation in patients with TB and HIV coinfection [2]; however, many significant drug–drug interactions (DDIs) exist, particularly with rifamycins [3]. Additionally, a major milestone in the advancement of HIV care for people with HIV was the approval of long-acting (LA) injectable cabotegravir/rilpivirine (CAB/RPV) [4]. As ART advancements focus on LA treatments and global TB incidence remains relatively unchanged, there is a need to assess the potential DDIs with co-administration of these therapies. Here, we present a real-world case of LA CAB/RPV co-administered with intravenous (IV) rifampin (RIF) in a patient with advanced HIV and multiple opportunistic infections.

## CLINICAL CASE

**Figure 1.**
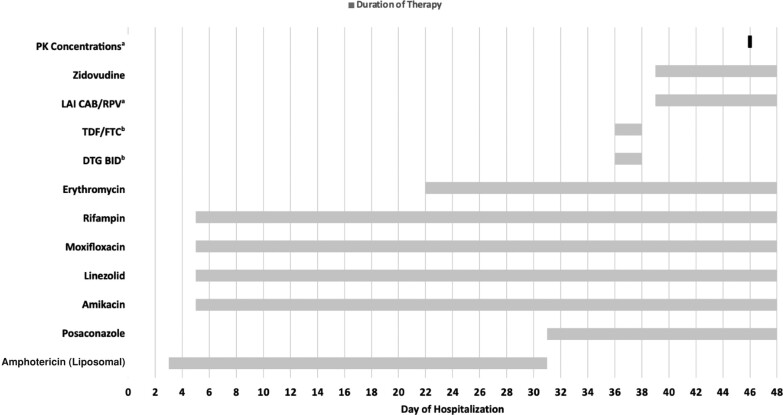
Anti-infective and pharmacokinetic concentration time course. ^a^LA CAB/RPV was administered on hospital day #39 at 1800, PK concentrations obtained on hospital day #46 at 1000. ^b^Both DTG and TDF/FTC were administered via nasogastric tube as a trial to determine if the patient could tolerate enteral medications; all other medications were administered parenterally. Abbreviations: BID, twice daily; DTG, dolutegravir; LAI CAB/RPV, long-acting injectable cabotegravir/rilpivirine; PK, pharmacokinetic; TDF/FTC, tenofovir disoproxil fumarate/emtricitabine.

A 49-year-old Sudanese male (weight = 66.1 kg) with no reported past medical history presented to the emergency department with several weeks of progressive encephalopathy and was found to be in septic shock with multi-organ failure, including respiratory failure requiring mechanical ventilation and acute renal failure requiring continuous renal replacement therapy. He was admitted to the intensive care unit, and a broad infectious workup revealed a new diagnosis of advanced HIV (HIV RNA = 470 000 copies/mL; CD4 = 13 cells/mm^3^; CD4% = 15.1%), miliary TB without evidence of TB meningitis, disseminated histoplasmosis, and fungal endophthalmitis. HIV-1 Drug Resistance RNA genotype (Next Generation Sequencing) found pan-sensitive/wild-type HIV virus. There was no evidence of active hepatitis B infection. His hospitalization was further complicated by severe bowel dysmotility. Gastroenterology was consulted and determined that the gastrointestinal tract was not usable for either nutritional or pharmacological therapies. Thus, a completely parenteral regimen was required for all therapies. After consultation with national TB colleagues at the Heartland National Tuberculosis Center [5], the patient was initially started on an antitubercular regimen of IV RIF, amikacin, moxifloxacin, and linezolid, in addition to liposomal amphotericin, for his disseminated histoplasmosis and fungal endophthalmitis. The liposomal amphotericin was eventually transitioned to posaconazole due to profound pancytopenia. Gastroenterology also recommended the addition of IV erythromycin to assist with his unresolving severe bowel dysmotility. Figure 1 illustrates the timeline of the anti-infective course of therapy.

Administration of ART was initially held while awaiting baseline HIV genotype results and ruling out TB meningitis. A shared decision was made to initiate ART after an interdisciplinary goals of care discussion. Despite the bowel dysmotility, the patient was trialed on a conventional ART regimen of dolutegravir 50 mg twice daily plus emtricitabine/tenofovir disoproxil fumarate 200/300 mg once daily via nasogastric tube. However, this resulted in intolerability, with severe emesis following each administration (Figure 1). Therefore, an unconventional ART regimen of LA 600 mg CAB plus 900 mg RPV given once intramuscularly, plus IV 1 mg/kg zidovudine administered every 4 hours, was initiated to avoid further delays in ART pending gut function recovery. Due to the complexity of the case and the potential for multiple DDIs with the anti-infective and ART regimen, we obtained plasma to measure concentrations of CAB/RPV. We did not measure zidovudine concentrations due to dose modifications not being warranted when co-administered with rifampin [6].

Plasma concentrations of CAB/RPV were obtained 7 days (Figure 1) post–loading dose of 600 mg intramuscular CAB and 900 mg intramuscular RPV. The concentrations were: CAB, 455 ng/mL; RPV, below the limit of quantitation (BLQ; LLOQ = 5 ng/mL). Unfortunately, the patient died 48 days after initial presentation due to multi-organ failure, so additional drug concentrations could not be collected. Furthermore, the pharmacokinetic concentrations were not available until after the patient died, so we were unable to make any dose adjustments based on the results.

## DISCUSSION

To our knowledge, there are limited real-world data on LA CAB/RPV concentrations when combined with rifamycins. Previous studies assessing oral CAB and RIF demonstrated a 59% reduction in CAB exposure, as measured by the area under the concentration time curve (AUC_0-∞_), while oral RPV with RIF had an 80% AUC reduction [3, 7–9]. More recently, pharmacokinetic modeling demonstrated a potential 40%–60% decrease in LA CAB AUC and a 40%–80% decrease in LA RPV AUC when co-administered with RIF [7–9]. It is relevant to note the difference in potential mechanisms of DDIs based on their route of administration and metabolism. Administration of LA CAB/RPV intramuscularly allows the drug to bypass the gastrointestinal tract, thus avoiding absorption-related DDIs, such as changes in gastric pH or drug chelation [9]. However, LA CAB/RPV still remains susceptible to drug metabolism–related DDIs. The primary mechanism of metabolism for CAB is by uridine diphosphate-glucuronosyl transferase 1A1 (UGT1A1) while RPV is metabolized by cytochrome P450 3A4 (CYP3A4) [8]. As RIF is a potent inducer of both CYP3A4 and UGT1A1, this corresponded with the observed concentrations of CAB and RPV in this patient, with RIF impacting RPV exposure to a greater extent compared with CAB [7–9]. Our patient received IV RIF, which is interchangeable with oral RIF (1:1 dose conversion); therefore, these results are expected to be similar to oral RIF.

The CAB concentration of 455 ng/mL 7 days after the LA CAB/RPV intramuscular loading dose was ∼2.7 times the protein-adjusted IC_90_ (PA-IC_90_) [10], which falls below the proposed concentration–response target of 4 times the PA-IC_90_ [7]. Previous pharmacokinetic parameters of LA CAB suggest that 7 days post–loading dose, peak concentration levels of intramuscular CAB result in a mean max concentration of 4 mcg/mL (90% CI, 2.3–6.8 mcg/mL) [9]. Based on these results, future areas of research include investigating whether dose escalation of LA CAB in combination with RIF may result in increased CAB concentrations. However, LA CAB does not demonstrate linear pharmacokinetics [7, 8]. Therefore, based on this case, administering a second initial dose of LA CAB may only have modest additional pharmacokinetic benefit, which may not reach the target of 4 times the PA-IC_90_. Importantly, the RPV concentration was BLQ after the intramuscular loading dose. Previous pharmacokinetic parameters for LA RPV suggest that peak concentration levels of intramuscular RPV occur 3–4 days postdose, with a median max concentration of 133 ng/mL (90% CI, 77.8–223 ng/mL) [9]. While it may have been beneficial to obtain an RPV concentration 3–4 days post–initial loading dose, due to the patient's hemodynamic instability and pancytopenia, we elected to only collect 1 set of samples. As confirmed by these concentrations, we recognized that RIF was more likely to impact RPV compared with CAB, which is why we also administered IV zidovudine.

As shown in Figure 1, the patient's anti-infective course of therapy was complex due to his severe bowel dysmotility, the need to treat his multiple opportunistic infections, and the potential for multiple DDIs. We recognize that rifabutin co-administration would have been a TB treatment option with less potential for DDIs than RIF, as it is a moderate CYP3A4 inducer [9, 11]; however, there were no available IV formulations of rifabutin available in the United States. Co-administration of rifabutin with oral CAB resulted in only a 17% decrease in max concentration and an AUC reduction of 23%, which was determined to not be clinically meaningful [11]. Furthermore, the co-administration of oral RPV with rifabutin only reduced the AUC by 41%, and the authors concluded that DDIs with moderate CYP3A4 inducers may be managed by increasing the RPV dosage (ie, administration of oral rilpivirine 25 mg daily in conjunction with monthly LA CAB/RPV); however, the prescribing information for LA CAB/RPV recommends against co-administration with rifabutin [4, 7, 12]. Thus, there are currently no recommendations on how to manage DDIs with intramuscular RPV and moderate inducers. We also noted the potential DDIs for LA CAB/RPV with erythromycin and posaconazole; however, we would have expected these DDIs to have potentially increased the concentration of RPV via CYP3A4 inhibition [4]. Despite these potential interactions, the RPV concentrations demonstrated an overwhelming impact from RIF CYP3A4 induction in comparison with erythromycin and posaconazole CYP3A4 inhibition.

We recognize the unconventional nature of this treatment approach; however, our patient required a completely parenteral ART regimen due to a nonfunctional gastrointestinal tract. Other injectable ART therapies, such as lenacapivir, ibalizumab, and enfuvirtide, were not readily available at the time of this case (ie, not Food and Drug Administration approved or limited-distribution pharmacy access channels). As ART advancements lead to greater utilization of LA ART for treatment of people with HIV, there is a need to better understand the real-world implications of LA ART and DDIs. Additionally, this case highlights the potential utility of obtaining drug concentrations when there is concern for potential DDIs. We believe that this case further emphasizes the importance of assessing the real-world pharmacokinetic implications of LA ART when combined with rifamycins or other co-medications with induction properties.
